# Professorial Appointments and Changes

**Published:** 1848-11

**Authors:** 


*Professorial Appointments and Changes.*—Professors Mitchell, Gardener, Beatty and Burden of the faculty of the Philadelphia College of Medicine have resigned, and Drs. Savory, Kennedy and Vandyke appointed. The particular chairs held by the latter respectively are not mentioned in the Examiner, from which the above intelligence is taken.

Dr. Jaynes, Professor of Institutes in the Franklin Medical College has resigned

